# Effectiveness Of Supervision On Work Engagement And Turnover Intention Of Care Managers in Japan

**DOI:** 10.1093/geroni/igab046.3088

**Published:** 2021-12-17

**Authors:** Ryosuke HATA

**Affiliations:** Hokusei Gakuen University, Sapporo, Hokkaido, Japan

## Abstract

In Japan, care managers engage frail older adults to support their assisted living in long term care insurance system. However, due to the lack of some or all supervision, many care managers face problems such as low work engagement and high turnover rate. This study aims to examine what types of supervision have positive effects on work engagement and turnover intensions of care managers in Japan. The sample of 241 care managers were asked whether they have received individual supervision in the workplace (ISVW), individual supervision in the community (ISVC), group supervision in the workplace (GSVW), or group supervision in the community (GSVC). Independent samples t-tests and one-way ANOVAs were conducted to examine the effectiveness of each types of supervision on work engagement and turnover intension. T-tests showed that only GSVW was significantly related to work engagement (t=-2.06, p<0.05). Whereas, only ISVW had a significant effect on turnover intensions (t=2.37, p<0.05). One-way ANOVAs revealed that 28 care managers receiving GSV had significantly higher work engagement than 92 care managers who did not receive any SV (F=5.33, p<0.01). 40 care managers receiving both ISV and GSV showed significantly lower turnover intentions than 92 care managers who received neither ISV nor GSV (F=2.84, p<0.05). Since the results have implications for the importance of supervisions to enhance work engagement or to reduce turnover intension of care managers, a larger sample will need to confirm these effects.

